# Shear Bond Strength of a Universal Adhesive to Enamel at Immediate, 7-Day and 14-Day Intervals After 40% Hydrogen Peroxide Bleaching: An In Vitro Study

**DOI:** 10.3390/bioengineering13091038

**Published:** 2026-09-06

**Authors:** Sultan Binalrimal

**Affiliations:** Department of Restorative Dentistry, College of Medicine and Dentistry, Riyadh Elm University, Riyadh 11681, Saudi Arabia; sultan@riyadh.edu.sa; Tel.: +966-50-166-6554

**Keywords:** tooth bleaching, hydrogen peroxide, shear strength, composite resins, dental enamel, universal adhesive, adhesive–enamel interface

## Abstract

High-concentration in-office bleaching reduces resin–enamel bond strength, but the required waiting interval remains unresolved, and much evidence predates universal adhesives. This study evaluated the apparent shear bond strength (SBS) of Palfique Universal Bond (Tokuyama Dental, Tokyo, Japan; lot no. 162E01), in etch-and-rinse mode after 37% phosphoric acid etching, at three intervals after 40% hydrogen peroxide bleaching. Eighty human premolar slabs, stored in artificial saliva at 37 °C, were allocated to a 14-day unbleached reference or to bleached specimens bonded immediately, at 7 or at 14 days, with one of two composites (n = 10 per group). Experimental condition significantly affected SBS (*p* < 0.001); no statistically significant effect of composite material or material × condition interaction was detected. Pooled across composites, SBS fell below the reference (4.62 MPa) after immediate bonding (4.18 MPa), at 7 days (3.81 MPa), and at 14 days (4.04 MPa; all adjusted *p* ≤ 0.034). With approximately 80% power to detect 0.55 MPa against observed differences of 0.14–0.37 MPa, the interval comparisons are inconclusive rather than evidence of equivalence; lacking time-matched controls at earlier intervals and conventional absolute values, the findings are within-study comparative evidence. Against the time-matched 14-day unbleached reference, however, apparent SBS remained significantly lower at 14 days, and restoration to the unbleached reference level should not be assumed for the protocol tested.

## 1. Introduction

Tooth discoloration is among the most common aesthetic complaints in restorative dental practice, and vital tooth bleaching remains the most frequently requested and least invasive treatment for it [1]. Since the night-guard vital bleaching technique was popularized in 1989 [2], whitening protocols based on hydrogen peroxide (H_2_O_2_) or its precursor carbamide peroxide have diversified into at-home and in-office systems that differ considerably in concentration, application time, and mode of activation [3]. High-concentration hydrogen peroxide formulations, typically 35–40% H_2_O_2_ applied under professional supervision, are commonly used for in-office (power) bleaching and may expose enamel to a substantially greater peroxide challenge over a relatively short period than lower-concentration home-bleaching systems [3,4,5]. Such formulations are, moreover, typically applied in two to three consecutive applications within a single chairside visit in order to accelerate the colour change [6]. The present study is concerned specifically with this high-concentration, short-duration exposure rather than with the much larger literature on low-concentration at-home bleaching.

Because the reactive oxygen species generated from H_2_O_2_ are not selective for pigment molecules, they also act on the dental hard tissues themselves; increased surface roughness, disruption of the enamel prism architecture and reduced microhardness have been reported, in a concentration- and time-dependent manner [4,5]. Of direct clinical relevance, bleaching has been consistently associated with reduced bond strength between resin composite and previously bleached enamel [7], with reductions of 25–60% reported when bonding is performed immediately [8]. Two overlapping mechanisms have been proposed to account for this: residual peroxide and entrapped oxygen inhibiting free-radical polymerization of resin monomers, and oxidative alteration of the enamel surface reducing the quality of micromechanical retention. Both are plausible, but neither was measured in the present work, and they are referred to throughout as proposed rather than established mechanisms.

Clinicians are therefore widely advised to delay adhesive restorative procedures after bleaching, but recommended post-bleaching bonding intervals vary from as little as 24 h to as long as three weeks [9,10]. The reported time course is similarly inconsistent: some investigators found SBS statistically comparable to unbleached controls within 1–2 weeks [10,11,12], others found no significant effect of elapsed time when lower bleaching concentrations were used [13], and orthodontic-bonding studies using high-concentration H_2_O_2_ have reported that bond strength can remain measurably compromised 14 days after bleaching [14], although others using a comparable concentration have judged a 14-day delay sufficient while a 7-day delay was not [15]. A systematic review and meta-analysis confirmed that vital bleaching significantly reduces bond strength to enamel and dentin but was unable to resolve the optimal interval, largely because of heterogeneity in peroxide concentration, adhesive chemistry, and storage protocol between primary studies [16]. Proposed alternatives to waiting, including mechanical removal of the outer 0.3–0.5 mm of bleached enamel before bonding [17] and topical antioxidant or remineralizing pretreatment [9,18,19,20,21,22,23], both add clinical steps that many practitioners would prefer to avoid.

Much of the evidence informing post-bleaching bonding intervals was generated using earlier adhesive systems, while evidence specifically addressing contemporary universal adhesives under high-concentration in-office bleaching conditions remains limited. Universal (multi-mode) adhesives are now among the most widely used bonding systems in restorative practice [24], and on enamel they are most commonly applied with prior phosphoric-acid etching, an approach shown to yield higher bond strengths than the self-etch mode on both sound and bleached enamel [25]. Whether waiting intervals derived largely from studies using earlier adhesive systems remain applicable to contemporary universal adhesives used in standardized etch-and-rinse protocols after high-concentration in-office bleaching remains insufficiently established; the published evaluation of universal adhesives on bleached enamel compared application modes but explicitly placed different post-bleaching intervals outside its scope [25].

This evidence gap, together with the rationale for the selected adhesive and post-bleaching intervals, can be summarized in three points. First, no prior study was identified that combined a contemporary universal adhesive with prespecified immediate, 7-day and 14-day post-bleaching bonding intervals after high-concentration in-office bleaching. The available evaluation of universal adhesives on bleached enamel compared application modes at a single time point [25], and the recent demonstration that a dual-cure activator improves universal-adhesive bonding to bleached enamel likewise tested a single interval [26].

Second, the adhesives examined in that literature are predominantly light-cured single-bottle systems, whereas Palfique Universal Bond is a two-component adhesive that polymerizes through a self-curing radical mechanism without a separate light-curing step and without a dual-cure activator. Because the leading proposed mechanism of impaired bonding to bleached enamel is inhibition of free-radical polymerization by residual peroxide and entrapped oxygen, an adhesive relying on a self-curing radical system is mechanistically relevant to that proposed interference, and it has recently been reported to yield the lowest bond strength to unbleached enamel among three universal adhesives tested [26].

Third, intervals of 7 and 14 days were selected because they bracket the waiting period most commonly recommended to clinicians and supported by current meta-analytic evidence [16]. Fourteen days is the point at which pooled analyses no longer detect an effect of bleaching, and it is therefore the interval at which a persistent deficit would be most informative; the immediate condition was retained as the earliest post-bleaching interval.

From a restorative bioengineering perspective, the interaction between peroxide-induced changes in the enamel substrate and the performance of an adhesive–enamel interface represents a material–tissue interaction that may influence the mechanical reliability of subsequent restorative procedures. Characterizing how that interface behaves after a defined oxidative challenge, and whether its behaviour changes over a clinically relevant waiting period, is therefore directly relevant to the selection and sequencing of restorative materials.

Accordingly, the present study evaluated whether immediate bonding and bonding after 7 and 14 days following 40% H_2_O_2_ bleaching resulted in shear bond strength different from an unbleached enamel reference when a contemporary universal adhesive was applied using an etch-and-rinse protocol. By examining the adhesive–enamel interface under a standardized oxidative challenge, the study aimed to provide evidence relevant to restorative material performance following in-office bleaching. Two contemporary composite systems (Estelite Sigma Quick and OMNICHROMA) (Tokuyama Dental Corporation, Tokyo, Japan) were included as a robustness check on the bleaching effect, and a secondary objective was to characterize the mode of failure at each experimental condition by stereomicroscopic fractography.

Three null hypotheses were tested, mirroring the statistical model: (H0_1_) the experimental condition would not affect enamel SBS; (H0_2_) the resin composite material would not affect SBS; and (H0_3_) there would be no interaction between experimental condition and composite material.

## 2. Materials and Methods

### 2.1. Study Design and Control Structure

**Figure 1:**
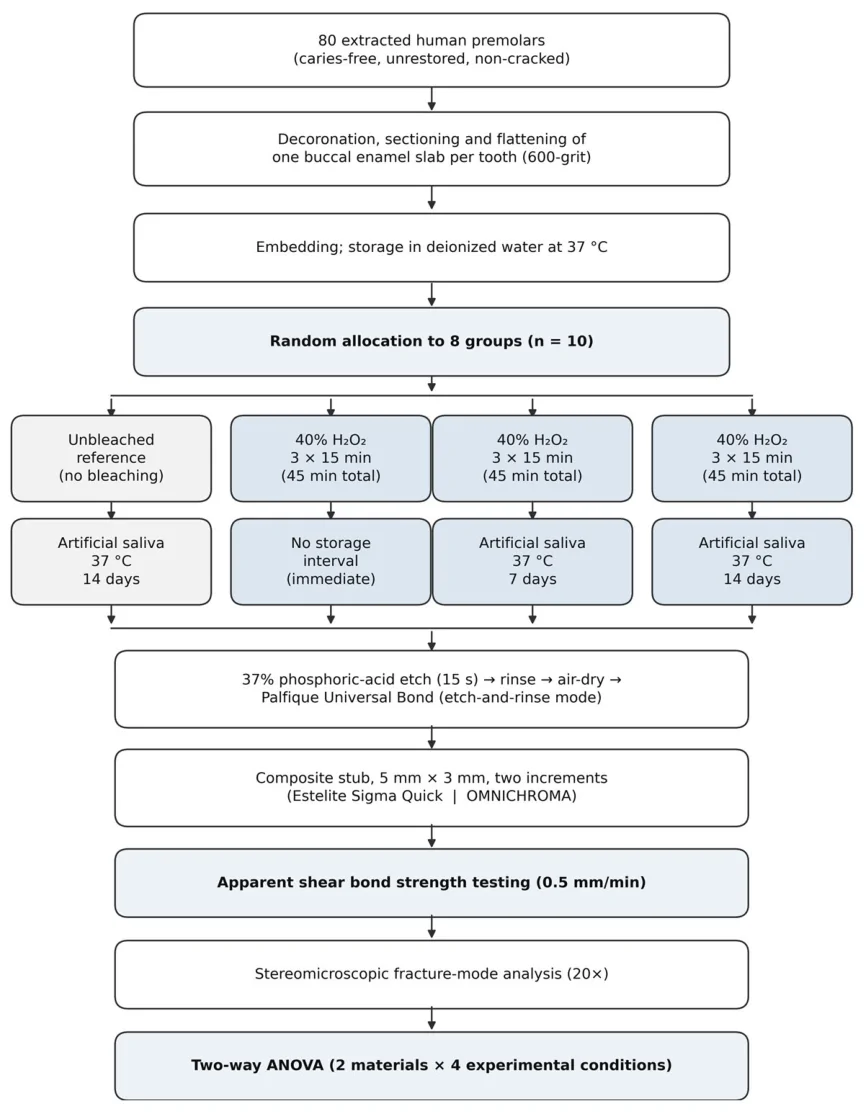
Experimental workflow. Eighty enamel slabs were randomly allocated to eight groups (two composite materials × four experimental conditions; n = 10 per group). The four-level experimental-condition factor comprises one unbleached 14-day reference condition and three post-bleaching bonding intervals; unbleached time-matched controls at the immediate and 7-day intervals were not included.

This experimental laboratory (in vitro) study evaluated the apparent shear bond strength (SBS) of two resin composites, bonded to human enamel with a universal adhesive applied in etch-and-rinse mode, under four predefined experimental conditions. The four-level experimental-condition factor comprised an unbleached 14-day reference and three post-bleaching bonding intervals (immediate, 7 days, and 14 days) after in-office bleaching with 40% H_2_O_2_. The complete experimental workflow is summarized in Figure 1.

The study was designed around a single 14-day unbleached reference condition, so that the 14-day bleached specimens could be compared against unbleached enamel exposed to the same storage duration; that comparison is time-matched and is treated throughout as the most directly interpretable contrast. Unbleached controls were not included at the immediate and 7-day intervals, so the comparisons of those two conditions against the reference cannot separate an effect of bleaching from a possible effect of storage duration, and the design cannot characterize the kinetics of recovery after bleaching. Conclusions regarding temporal recovery are restricted accordingly.

A complete design crossing composite material (two levels), bleaching status (bleached versus unbleached) and storage interval (0, 7 and 14 days) would comprise 12 experimental groups and 120 specimens at n = 10 per group; the present study comprised eight groups within the available specimen pool. The design was constrained to that pool of 80 specimens and prioritized a time-matched comparison at 14 days, the waiting interval most commonly recommended clinically [16]; inclusion of unbleached controls at the immediate and 7-day intervals would have produced a stronger factorial design, and their absence is acknowledged as the principal design limitation. The experimental-condition factor is therefore interpreted as a comparison among the predefined experimental conditions rather than as a pure measure of temporal recovery after bleaching. The term “experimental condition” is used throughout for the four-level factor, and “post-bleaching bonding interval” is reserved for the three bleached levels (immediate, 7 days, 14 days).

Specimen preparation and adhesion testing were informed by the general principles of ISO/TS 11405:2015 [27] for testing adhesion to tooth structure; however, several protocol modifications were introduced to address the specific experimental objectives, and the protocol was therefore not formally ISO-compliant. The modifications were: (i) the composite stub was 5 mm high and 3 mm in diameter, giving a height-to-diameter ratio of 1.67 rather than the squatter configuration commonly adopted; (ii) the bleaching regimen departed from the manufacturer’s stated application time (Section 2.6); (iii) specimens were stored in artificial saliva rather than water during the post-bleaching interval; and (iv) shear testing was performed immediately after composite placement and removal of the mold, rather than after the 24 h of storage at 37 °C specified in the standard.

A standardized 5 mm-high cylindrical composite configuration was used for all groups in order to maintain identical specimen geometry across experimental conditions and to provide a stub of sufficient height for reproducible blade placement. This was a deliberate methodological choice; the configuration differs from conventional shear bond-testing geometries and is not intended to represent a clinically realistic restoration. Absolute SBS values were therefore interpreted as within-study comparative measurements rather than as transferable estimates of clinical bond strength. To support that framework, the same composite height, incremental placement, curing duration, light-curing unit, tip position and loading parameters were used for all specimens, so that variability associated with specimen geometry and curing conditions was standardized across groups.

Extracted, non-carious human premolars were used in preference to bovine enamel, which yields systematically different bond-strength values. All specimen preparation, bleaching, bonding and testing procedures were carried out by a single trained operator.

### 2.2. Sample Size

Sample size was estimated a priori using G*Power version 3.1 (Heinrich Heine University, Düsseldorf, Germany), selecting “F tests: ANOVA: Fixed effects, special, main effects and interactions”. The calculation was based on the primary main-effect comparison, namely the main effect of experimental condition (four levels; numerator df = 3) within a 2 (composite material) × 4 (experimental condition) design comprising eight cells, with α = 0.05, power (1 − β) = 0.80 and an anticipated effect size of f = 0.40 based on bond-strength differences reported in comparable post-bleaching studies [10,14]. This indicated a minimum total sample of 80 specimens, i.e., 10 per group; 80 slabs were therefore prepared. The calculation was not specifically powered to detect the material × experimental-condition interaction, and the within-material comparisons are correspondingly under-powered; the interaction and within-material analyses are accordingly reported as exploratory.

Because the a priori calculation targeted the main effect of experimental condition, a sensitivity analysis was performed on the ANOVA residual variance to establish the smallest differences the realized design could resolve. For pairwise comparisons among the three post-bleaching intervals (n = 20 per condition pooled across composites, Bonferroni-adjusted α = 0.0083), the design could detect a difference of approximately 0.55 MPa with 80% power; detecting a difference of 0.37 MPa would require approximately 44 specimens per condition. The 0.55 MPa value denotes the difference detectable with 80% power at the corrected significance level and is not a threshold below which differences cannot be detected; smaller differences may still reach significance at lower power, as the 0.44 MPa reference contrast did. The design likewise had limited sensitivity to small material × condition interactions. Comparisons among intervals and the interaction term are therefore interpreted as inconclusive rather than null, and no retrospective power values computed from the observed effects are reported, since such values are a monotone function of the corresponding *p*-values and add no information.

### 2.3. Specimen Selection and Preparation of Enamel Slabs

Eighty extracted maxillary and mandibular premolars, obtained from patients aged 22–50 years undergoing extraction for orthodontic or periodontal reasons, were used; the donor age range is reported descriptively and was determined by the available extraction pool rather than by a predefined selection criterion. Teeth were disinfected, stored for one week in 0.1% aqueous thymol solution, and used within six months of extraction. Sound permanent teeth were included only if they were free of caries, restorations, cracks, developmental or hypoplastic defects, visible wear facets on the buccal surface, and other structural anomalies; hypo- and hyper-mineralized teeth were excluded. Teeth were cleaned and polished with a rubber cup, pumice, and water at low speed, then rinsed with deionized water.

Each tooth was decoronated at the cemento-enamel junction using a diamond disc (Isomet, Buehler, Lake Bluff, IL, USA), and the crown was sectioned to produce one slab per tooth measuring approximately 4 × 4 × 3 mm from the buccal surface. The middle third of the buccal surface of each slab was flattened using a diamond disc under continuous air–water cooling and finished with 600-grit silicon carbide paper under running water, with grinding limited to the minimum required to obtain a flat bonding area. The bonding surface was monitored visually to ensure it remained entirely within enamel. This preparation prioritized standardization of the bonding surface across all specimens over preservation of the native aprismatic enamel morphology; the consequences of that choice are considered in the Discussion. Residual enamel thickness after flattening was not measured instrumentally.

The slabs were embedded in auto-polymerizing clear orthodontic resin in a 10-cm-diameter PVC mold, leaving the flattened enamel surface exposed and flush with the surrounding resin, and were stored submerged in deionized water at 37 °C until allocation to the experimental groups following specimen preparation.

### 2.4. Group Allocation and Study Groups

Allocation was performed at the specimen level after specimen preparation and eligibility screening and before any experimental treatment, in the sequence tooth collection → preparation → eligibility assessment → group allocation → bleaching or reference storage → bonding → testing (Figure 1). Each tooth contributed one enamel slab, each slab constituted one independent specimen, and the 80 slabs were randomly allocated to eight experimental groups of 10 specimens.

Four groups were bonded with Estelite Sigma Quick (G1–G4) and four with OMNICHROMA (B1–B4). Within each material, one group served as the unbleached 14-day reference condition (G1, B1) and three were bleached with 40% H_2_O_2_ and bonded either immediately (G2, B2), after 7 days (G3, B3), or after 14 days (G4, B4). The two composites were included as a robustness check on the bleaching effect rather than as a co-equal research question: Estelite Sigma Quick is a conventional shade-based supra-nano filled composite and OMNICHROMA is a single-shade material that generates colour by structural light scattering, while the adhesive was held constant.

### 2.5. Storage Medium and Conditions

Specimens were stored fully immersed in freshly prepared Fusayama–Meyer artificial saliva (pH approximately 6.8), with each specimen stored separately in 10 mL of medium in an individually sealed 20 mL polypropylene screw-cap container. Thus, the nominal medium volume per specimen and the storage environment were standardized across groups. Storage was maintained continuously at 37 °C in an incubator, with the temperature checked daily using a calibrated digital thermometer, and specimens were transferred to freshly prepared artificial saliva every 48 h. Artificial saliva was used in preference to deionized water to approximate the ionic and remineralizing environment of the oral cavity during the post-bleaching interval.

### 2.6. Bleaching Protocol

A commercially available 40% H_2_O_2_ in-office bleaching gel (Opalescence Boost PF 40%, Ultradent Products Inc., South Jordan, UT, USA; lot no. BJYL4) was used. Slabs first underwent prophylaxis with a rubber cup and pumice and were rinsed and lightly air-dried. The gel was then applied to the flattened enamel surface of groups G2–G4 and B2–B4.

A standardized three-application protocol of 15 min per application was used to provide a controlled cumulative peroxide exposure across specimens. At the end of each application, the gel was removed, the enamel surface was thoroughly rinsed with water, and fresh gel was applied for the subsequent cycle, giving a cumulative peroxide contact time of 45 min. This protocol is described as a standardized experimental exposure model rather than as an exact reproduction of the manufacturer’s clinical instructions. The manufacturer recommends 20 min per application, with two to three applications and a maximum of three applications in a single visit [6]. Accordingly, the difference in application duration is acknowledged as a limitation on the clinical transferability of the exposure model.

### 2.7. Post-Bleaching Storage and Experimental Timeline

The timeline for each experimental condition was as follows. Unbleached reference specimens (G1, B1) were stored in artificial saliva at 37 °C for 14 days without bleaching and were then bonded and tested. Immediate-bonding specimens (G2, B2) were bleached, rinsed, and prepared for bonding and testing directly after the final application, without any intervening storage period. Specimens in the 7-day groups (G3, B3) were bleached, rinsed, stored in artificial saliva at 37 °C for 7 days, and then bonded and tested. Specimens in the 14-day groups (G4, B4) were bleached, rinsed, stored in artificial saliva at 37 °C for 14 days, and then bonded and tested. All storage conditions, container type, medium volume, and renewal frequency were identical across the reference, 7-day, and 14-day conditions.

### 2.8. Adhesive Application

A single universal adhesive, Palfique Universal Bond (Tokuyama Dental, Tokyo, Japan; lot no. 162E01), was used throughout, applied in etch-and-rinse mode. Enamel was etched with 37% phosphoric acid gel for 15 s, rinsed for 15 s, and gently air-dried. The same etching procedure and timing were used for all specimens to standardize enamel pretreatment. The two adhesive components (Bond A and Bond B) were dispensed and mixed in equal volumes, applied to the etched enamel with a disposable applicator, left undisturbed for 10 s, and then dried with a stream of oil-free air until the solvent had evaporated and the surface no longer moved. In accordance with the manufacturer’s instructions, the adhesive was not light-cured as a separate step before composite placement: Palfique Universal Bond polymerizes through a self-curing mechanism and is compatible with light-, self- and dual-cured composites without a separate activator or dedicated adhesive curing cycle. Because a single adhesive was evaluated in a single application mode, the findings are referred to throughout as applying to the tested universal adhesive in etch-and-rinse mode and are not generalized to universal adhesives as a class or to their self-etch and selective-etch modes.

### 2.9. Composite Placement and Light Curing

Composite stubs of Estelite Sigma Quick (Tokuyama Dental, Tokyo, Japan; lot no. E8103) or OMNICHROMA (Tokuyama Dental, Tokyo, Japan; lot no. 1701S2) were built in two successive increments within a Teflon mould (5 mm height × 3 mm diameter) placed over the enamel slab, giving a bonded surface area of 7.07 mm^2^. The 5 mm stub height was selected to provide a specimen tall enough for reproducible positioning of the shear blade and identical across all groups.

Each increment was light-cured for 20 s with an LED curing unit (Bluephase N, Ivoclar Vivadent, Schaan, Liechtenstein) at a measured output irradiance of 1500 mW/cm^2^, with the light tip held perpendicular to and in direct contact with the upper rim of the mould. Output irradiance was verified at the light tip before the experiment and monitored periodically during the experiment using a Bluephase Meter II radiometer (Ivoclar Vivadent). Because the composite stub was 5 mm high, the irradiance reaching the adhesive interface would have been lower than the value measured at the tip; the reported irradiance therefore represents the output measured at the curing-light tip rather than the irradiance delivered to the adhesive interface. The mould was removed before testing.

### 2.10. Shear Bond Strength Testing

Shear testing was carried out immediately after removal of the mould; bonded specimens were not stored between composite placement and testing, and were kept hydrated and tested at room temperature. Testing was performed on a universal testing machine (Model 4301, Instron, Canton, MA, USA) at a crosshead speed of 0.5 mm/min until failure, with a chisel-shaped blade loaded in a single direction parallel to the bonded interface and positioned as close as possible to it, perpendicular to the long axis of the composite stub. Load at failure was recorded in newtons using Bluehill software (Instron, Norwood, MA, USA) and converted to SBS in MPa by dividing by the bonded surface area. Each specimen contributed exactly one SBS value to the statistical analysis, and the specimen was therefore the statistical unit throughout. Because the specimen configuration produces a complex rather than a purely shear stress state at the interface, the measurement is referred to as apparent shear bond strength.

### 2.11. Fracture-Mode Analysis

After testing, debonded enamel surfaces were examined under a light stereomicroscope (Nikon SMZ-10, Tokyo, Japan) at 20× magnification. Fracture mode was classified operationally as follows: adhesive failure was defined as separation at the enamel–adhesive interface with no substrate or composite fracture; cohesive failure in resin was defined as fracture occurring wholly within the composite stub; cohesive failure in enamel was defined as fracture occurring within the enamel substrate; and mixed failure was defined as any combination of interfacial separation with cohesive fracture of resin or enamel within the same bonded area. Classification was performed by a single examiner blinded to group allocation; no second examiner assessed the specimens, and no formal intra-examiner reliability assessment was undertaken.

### 2.12. Statistical Analysis

Descriptive statistics (mean ± standard deviation, with 95% confidence intervals [CI]) were calculated for all groups, and reductions relative to the unbleached reference condition were additionally expressed as percentages to permit comparison with studies using different specimen configurations. Distributional assumptions were assessed using the Shapiro–Wilk test and homogeneity of variance using Levene’s test. No statistically significant evidence against normality or against homogeneity of variances was detected (*p* > 0.05); with 10 specimens per cell, these tests have limited power, so they are reported as an assessment of, rather than as proof of, the model assumptions.

A two-way analysis of variance (ANOVA) was fitted as a 2 (composite material) × 4 (experimental condition) factorial model, with partial eta-squared (η^2^_p_) reported as a measure of effect size for both main effects and the interaction. The second factor is described as experimental condition, not as time or post-bleaching time, because its four levels comprise one unbleached reference and three bleached conditions; a significant effect of this factor therefore indicates that the predefined experimental conditions differ and does not by itself identify elapsed time since bleaching as the cause of the difference.

Experimental conditions were compared using estimated marginal means from the factorial model, averaged across composite material, with Bonferroni correction across the six pairwise contrasts; presentation of the marginal condition effect did not depend on the interaction result. The material × condition interaction was examined separately; because the study had limited sensitivity to small interaction effects, a non-significant interaction was not interpreted as evidence that the two materials responded equivalently. Mean differences are reported with Bonferroni-adjusted 95% CIs derived from the ANOVA error term. The three comparisons of primary interest, defined in advance, were each post-bleaching bonding interval against the unbleached reference condition (immediate versus reference, 7 days versus reference, 14 days versus reference); the three comparisons among post-bleaching intervals are reported as secondary. Within-material comparisons are reported separately as exploratory analyses.

The distribution of fracture modes was compared using the Fisher–Freeman–Halton exact test across all eight groups; an exact test was chosen because the expected cell frequencies were small and several failure categories were sparsely populated, which would invalidate a chi-square approximation. Analyses were performed using IBM SPSS Statistics version 29.0 (IBM Corp., Armonk, NY, USA), with *p* < 0.05 considered statistically significant.

### 2.13. Ethical Approval

Ethical approval for the study was obtained from the Dental Research Institute, Riyadh Elm University, Riyadh, Saudi Arabia (IRB approval no. FPGRP/2021/644/660/648; registration no. FPGRP/2021/644/660; date of approval: 2 September 2022). Laboratory procedures were performed at the Dental Biomaterial Research Laboratory, King Saud University, Riyadh, Saudi Arabia. Donors gave informed consent for the use of their extracted teeth in research.

## 3. Results

**Figure 2:**
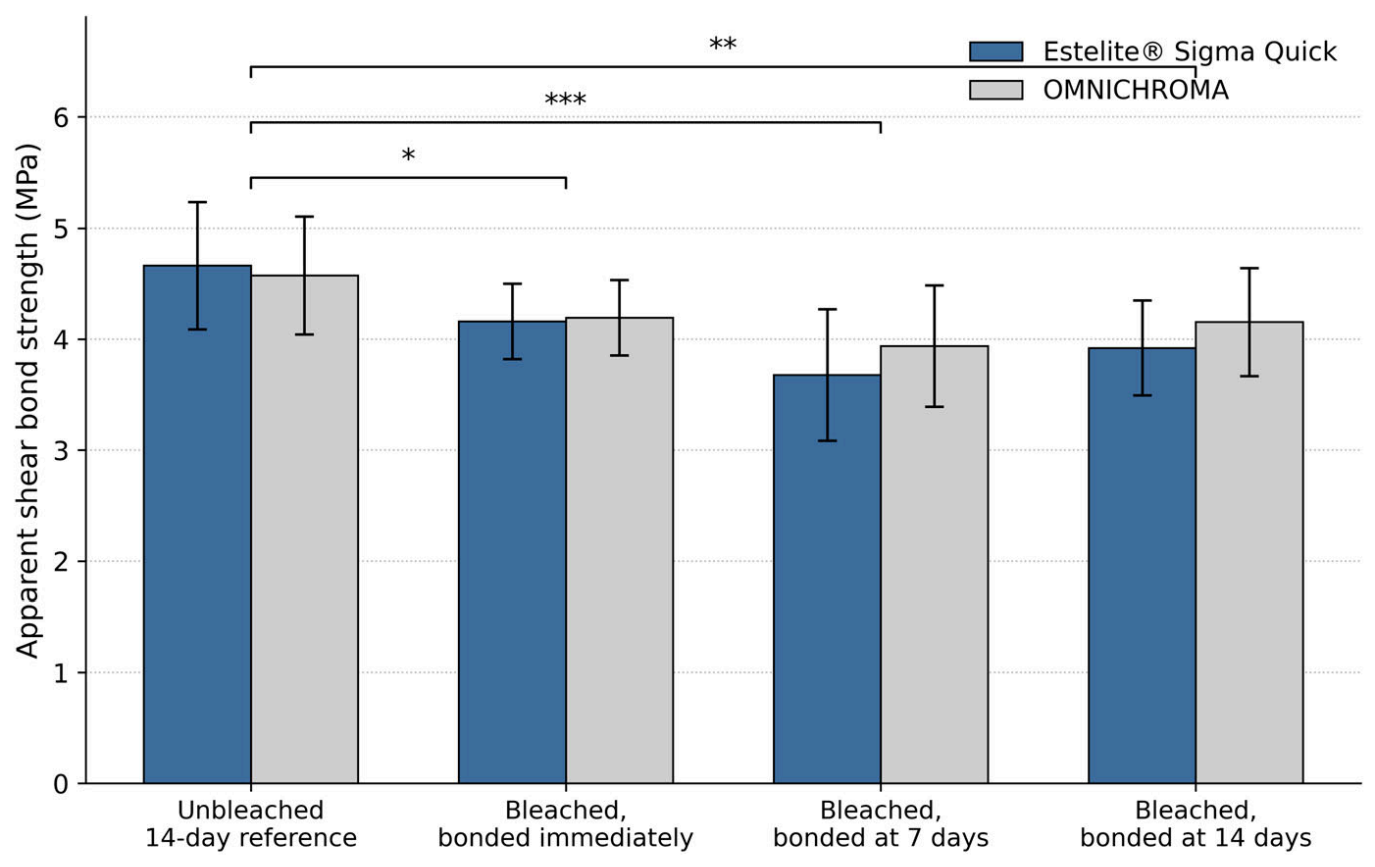
Mean apparent shear bond strength (MPa) of Estelite Sigma Quick and OMNICHROMA composite to enamel for each experimental condition (n = 10 per group). Error bars represent one standard deviation. Brackets indicate Bonferroni-corrected comparisons against the unbleached 14-day reference condition for experimental conditions pooled across both composite materials (* *p* < 0.05; ** *p* < 0.01; *** *p* < 0.001).

All 80 specimens were tested and included in the analysis; no specimen failed prematurely during handling or storage. Mean SBS values for each of the eight groups are presented in Table 1 and Figure 2. The principal finding was that mean SBS was highest in the unbleached 14-day reference condition and lower in all three bleached conditions, and that each bleached condition differed significantly from the reference (Table 2). Pooled across composites, the reduction relative to the reference was 0.44 MPa (9.6%) for immediate bonding (95% CI 0.02–0.86; *p* = 0.034), 0.81 MPa (17.5%) at 7 days (95% CI 0.39–1.23; *p* < 0.001) and 0.58 MPa (12.6%) at 14 days (95% CI 0.16–1.00; *p* = 0.002).

None of the three post-bleaching bonding intervals differed significantly from one another: immediate versus 7 days, mean difference 0.37 MPa (95% CI −0.05 to 0.79; *p* = 0.120); immediate versus 14 days, 0.14 MPa (95% CI −0.28 to 0.56; *p* = 1.000); and 7 days versus 14 days, −0.23 MPa (95% CI −0.65 to 0.19; *p* = 0.861). The study had approximately 80% power to detect a difference of 0.55 MPa (Section 2.2), whereas the observed differences were 0.14–0.37 MPa; their non-significance is therefore inconclusive and is not evidence that the intervals are equivalent.

Two-way ANOVA (Table 3) showed a significant effect of experimental condition on SBS (F_3,72_ = 9.70, *p* < 0.001; η^2^_p_ = 0.288), whereas no statistically significant main effect of composite material was detected (F_1,72_ = 1.00, *p* = 0.320; η^2^_p_ = 0.014). No statistically significant material × condition interaction was detected (F_3,72_ = 0.58, *p* = 0.633; η^2^_p_ = 0.023). Experimental conditions were summarized across the two composite materials using estimated marginal means from the factorial model (Table 2). H0_1_ was rejected, whereas there was insufficient evidence to reject H0_2_ or H0_3_.

Exploratory within-material comparisons were consistent with the pooled analysis. Among the Estelite Sigma Quick groups, the reference condition (G1) differed significantly from the 7-day (G3; *p* < 0.001) and 14-day groups (G4; *p* = 0.012), whereas G2 did not differ from G1 (*p* = 0.179) or from G3 and G4 (*p* > 0.05). Among the OMNICHROMA groups, only B1 versus B3 reached significance (*p* = 0.034). These comparisons, each based on 10 specimens per cell, were underpowered relative to the pooled analysis and are reported as exploratory.

**Figure 3:**
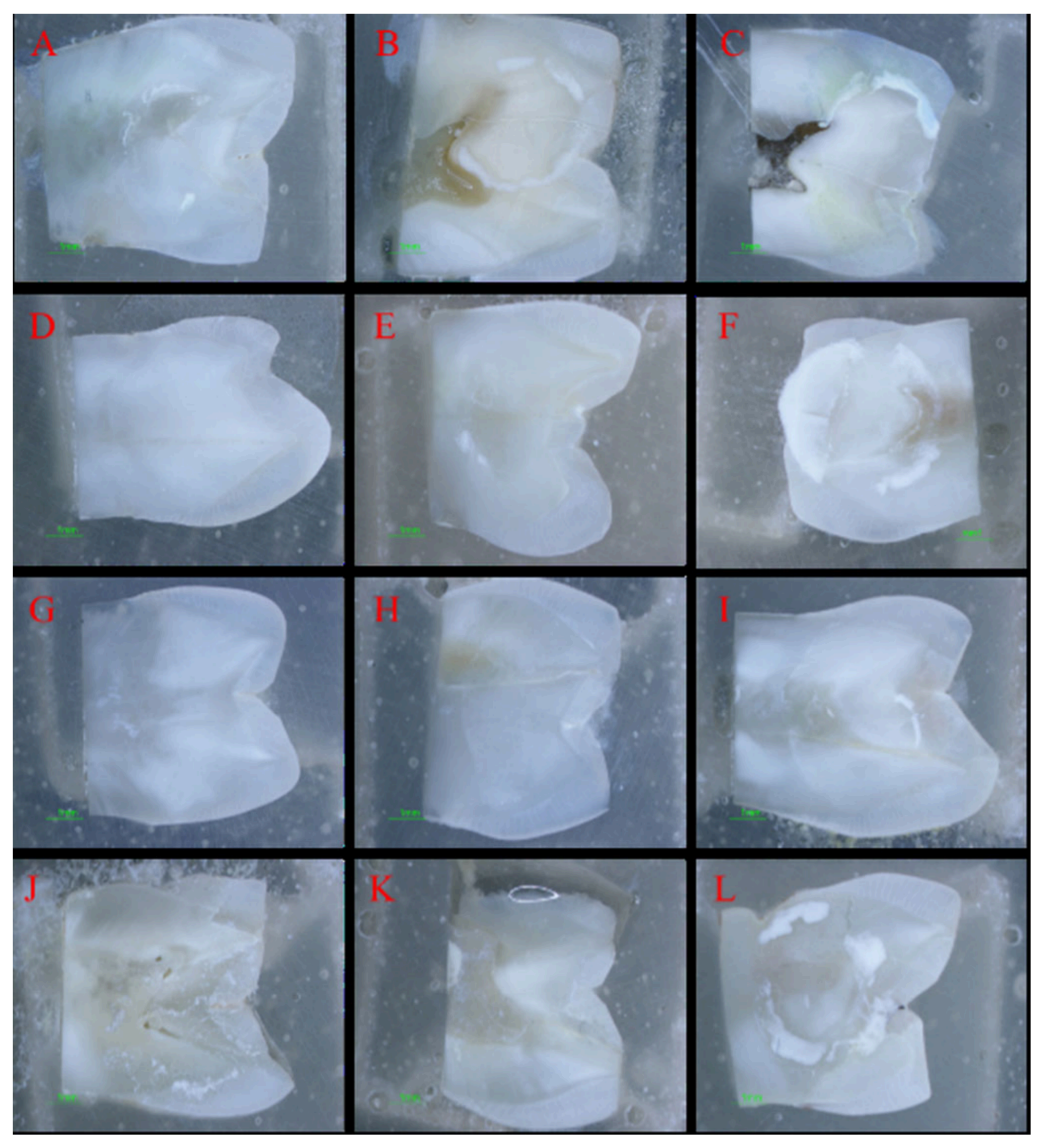
Stereomicroscopic images of debonded enamel surfaces bonded with Estelite Sigma Quick composite (green scale bar = 1 mm; original magnification 20×). (**A**–**C**) Adhesive, cohesive-in-resin, and mixed failure, respectively, in unbleached specimens stored in artificial saliva at 37 °C for 14 days (G1). (**D**,**E**) Adhesive failure and (**F**) cohesive-in-resin failure after bleaching with immediate bonding (G2). (**G**–**I**) Adhesive failure after bleaching with bonding at 7 days (G3). (**J**–**L**) Adhesive failure after bleaching with bonding at 14 days (G4).

**Figure 4:**
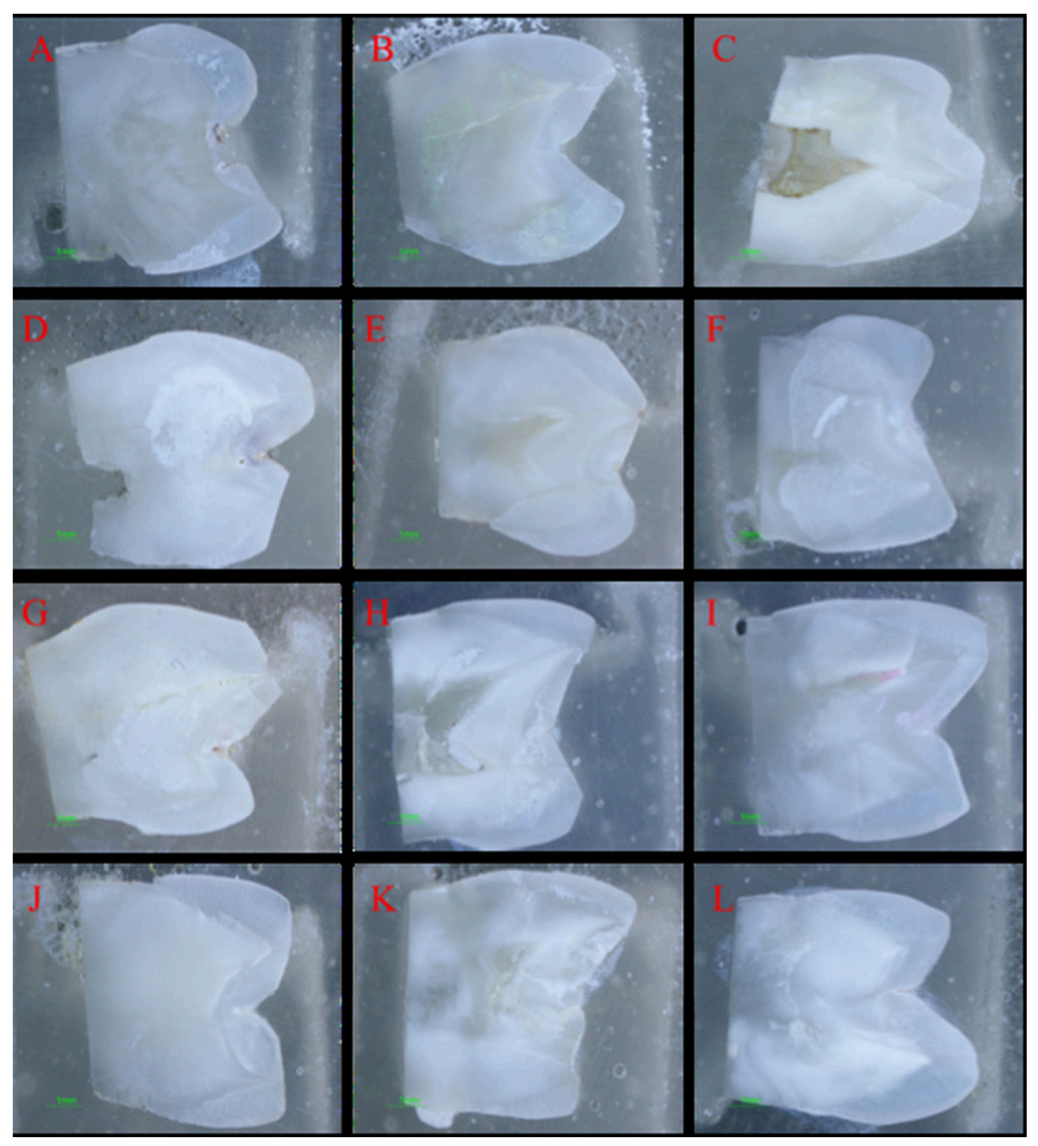
Stereomicroscopic images of debonded enamel surfaces bonded with OMNICHROMA composite (green scale bar = 1 mm; original magnification 20×). (**A**–**C**) Adhesive, cohesive-in-resin, and mixed failure in the unbleached 14-day reference group (B1). (**D**–**F**) Adhesive, cohesive-in-resin, and mixed failure after bleaching with immediate bonding (B2). (**G**,**H**) Adhesive failure and (**I**) cohesive-in-resin failure after bleaching with bonding at 7 days (B3). (**J**,**K**) Adhesive failure and (**L**) mixed failure after bleaching with bonding at 14 days (B4).

Fracture-mode distributions are presented in Table 4. Across all eight groups, the distribution did not differ significantly (Fisher–Freeman–Halton exact test, *p* = 0.377). Descriptively, adhesive failure occurred in 38/40 (95%) specimens in the 7- and 14-day bleached groups, compared with 13/20 (65%) in the 14-day unbleached reference groups. This comparison was not prespecified and is reported descriptively only. No cohesive failure within enamel was recorded in any group. Representative images of the debonded surfaces are shown in Figure 3 and Figure 4.

## 4. Discussion

Two statements can be drawn from these data, and it is worth separating them at the outset. What the study establishes is that, against a time-matched unbleached reference stored for the same 14 days under identical conditions, apparent enamel bond strength remained significantly lower 14 days after bleaching with 40% hydrogen peroxide (mean difference 0.58 MPa; adjusted *p* = 0.002). What it does not establish is the kinetics of that deficit: time-matched controls were unavailable for the immediate and 7-day conditions, the three post-bleaching intervals could not be distinguished from one another, and the specimen configuration precludes interpretation of the absolute values. The discussion that follows is framed accordingly.

Under the conditions of the present study, apparent SBS was lower in all three bleached conditions than in the 14-day unbleached reference. Most importantly, the 14-day bleached condition remained significantly below the time-matched 14-day unbleached reference. Differences among the immediate, 7-day, and 14-day bleached conditions were not statistically significant; these contrasts were, however, smaller than the difference the study had approximately 80% power to detect and therefore remain unresolved rather than demonstrably absent. H0_1_ was rejected. Expressed relative to the reference condition, the reduction was 9.6% for immediate bonding, 17.5% at 7 days, and 12.6% at 14 days.

However, interpretation of the temporal pattern requires caution, because the study included a single 14-day unbleached reference rather than time-matched unbleached controls. The unbleached reference specimens were tested after 14 days of storage, whereas the bleached specimens were bonded at 0, 7 and 14 days; the experimental factor analysed here therefore conflates bleaching status with storage duration. This affects the immediate-bonding comparison most directly, since those specimens are compared against a reference stored for a further 14 days. The consequence is that the present findings should be read as demonstrating a persistent reduction in SBS relative to the 14-day unbleached reference condition, and not as establishing the kinetics of bond-strength recovery after bleaching. Distinguishing a genuine bleaching effect from any effect of storage duration would require unbleached controls at every interval.

A second constraint concerns the absolute values obtained. The SBS recorded here (3.68–4.66 MPa) is substantially lower than the 20–35 MPa commonly reported for composite bonded to phosphoric-acid-etched enamel, and the specimen configuration is the most likely reason: a stub with a height-to-diameter ratio of 1.67 generates a considerable bending moment at the interface under shear loading; a 3 mm-diameter cylinder polymerized within a Teflon mold has a high configuration factor, so shrinkage stress pre-loads the bond; light reaching the interface through a 5 mm-deep mold is attenuated, so the degree of conversion at the interface, which was not measured here, may have been suboptimal; and testing immediately after mold removal allowed no post-cure maturation.

Although the absolute SBS values were therefore lower than those commonly reported using conventional specimen configurations, all experimental groups were evaluated using identical substrate preparation, adhesive application, composite geometry, curing conditions, and loading parameters. The principal inference of the study accordingly concerns relative differences among the predefined experimental conditions rather than absolute bond-strength values, and the absolute figures should not be compared numerically with other reports. A micro-shear or micro-tensile configuration would have provided a more conventional bonded geometry and greater comparability with published absolute bond-strength values; a bonded area of approximately 1 mm^2^ would also have reduced both the bending moment and the configuration factor that most plausibly account for the low values recorded here. Such a configuration is adopted in the planned follow-up.

Two considerations nonetheless support the interpretability of the present comparison. First, the confounding factors identified above are geometric and constant across all eight groups, which supports the validity of within-study comparison; it cannot be assumed, however, that a non-conventional geometry influences stress distribution and failure initiation entirely independently of substrate condition, and interpretation is accordingly restricted to the direction and relative magnitude of between-condition differences. Second, an independent study using a conventional shear configuration reported lower bond strength for Palfique Universal Bond than for two other universal adhesives tested [26]. Although differences in substrate and experimental design preclude attributing the low absolute values observed here directly to adhesive chemistry, this finding suggests that adhesive-specific performance may also contribute to the values obtained. No clinical threshold is claimed, and the conclusions remain restricted to relative comparison.

Consistent with this framing, the relative reductions observed here (9.6–17.5%) are smaller than the 25–60% reported in some earlier work [8]. The smaller relative reductions may partly reflect a floor effect: where the reference bond strength is itself low, less bond strength is available to be lost, and proportional reductions are correspondingly compressed. The present data therefore characterize the direction and the consistency of the bleaching effect rather than its clinical magnitude.

The mechanisms proposed for reduced bonding to bleached enamel fall into three groups. Chemically, residual peroxide and entrapped oxygen may inhibit free-radical polymerization at the interface. At the level of the substrate, oxidative alteration of the enamel surface may reduce surface energy, wettability, and micromechanical interlocking [28], with concurrent reductions in enamel microhardness [29] and in surface calcium and phosphorus content [30] reported after in-office bleaching. Experimentally, specimen geometry, light attenuation, and stress distribution may modulate the measured value independently of the substrate. None of these variables was measured here, and they are cited as context rather than as established mechanisms.

The present study was designed as a mechanical screening experiment and did not include nanoindentation, spectroscopic determination of the degree of conversion, or SEM and AFM characterization of the interface. The primary outcome was the mechanical bond-strength response at clinically relevant post-bleaching intervals; substrate-property measurements would help explain the observed differences but would not replace the mechanical endpoint. These analyses cannot be reconstructed retrospectively, because destructive shear testing irreversibly disrupted the original bonded interfaces: interfacial degree of conversion and intact interface morphology would require specimens characterized before testing, while dedicated nanoindentation and AFM characterization would require an additional specimen series. Their absence limits mechanistic interpretation.

To place that limitation in context, recent studies have directly characterized morphological, elemental and mechanical alterations in bleached enamel under related conditions: Saleem et al. [30] reported reduced calcium and phosphorus by EDX, increased roughness and grooving by AFM, and an approximately two-fold increase in indentation depth at constant load in bleached enamel, indicating reduced hardness and elastic modulus; Sivavong et al. [26] documented surface morphological alteration after bleaching by SEM and EDS in the same etch-and-rinse universal-adhesive configuration used here; and Buzatu et al. [29] reported concurrent chemical and microhardness changes in human enamel after in-office bleaching with the same 40% hydrogen peroxide product used here. Together, these supply a plausible substrate-level account of the reduction observed in the present work, but that linkage remains inferential here, and the degree of conversion at the adhesive interface in particular was not measured and cannot be inferred from bond strength alone. A combined mechanical and analytical design is identified as the priority for the next study.

The persistence of a deficit at 14 days is the principal point of departure from the current pooled evidence. Previous work can be grouped by the pattern reported. Studies describing a return toward unbleached values with elapsed time include Unlu et al. [11] and Cavalli et al. [12], and Miljkovic et al. [7] reported a similar return over shorter intervals under a different adhesive and protocol; Cura et al. [13], using a lower-concentration agent, found no significant effect of elapsed time at all. The systematic review and meta-analysis of Savian et al. [16] confirmed that vital bleaching significantly impairs bond strength to enamel and dentin overall but reported that this effect was no longer statistically detectable at two and three weeks, and recommended delaying restoration for at least two weeks.

At the other end of the range, orthodontic-bonding studies using comparable concentrations found bond strength still compromised at 14 days [14], a systematic review of bleaching and bracket adhesion likewise concluding that the evidence on the required delay remains inconsistent [31], prolonged bleaching has been reported to delay the return of adhesive performance [32], and de Almeida et al. [15] occupy an intermediate position, finding a 7-day delay insufficient but a 14-day delay adequate after 35% H_2_O_2_. The present results align more closely with studies reporting persistent impairment at 14 days, although differences in experimental design preclude direct comparison.

Direct comparison with the small universal-adhesive literature is now possible on three points. Oz and Kutuk [25] compared application modes and reported higher bond strengths in etch-and-rinse than in self-etch mode on both sound and bleached enamel, which is why the etch-and-rinse mode was adopted here; their design held elapsed time constant and therefore cannot be compared with the present interval data. Sivavong et al. [26] tested three universal adhesives on bleached bovine enamel in etch-and-rinse mode and found that bleaching significantly reduced bond strength in every adhesive group, with Palfique Universal Bond showing the lowest bond strength to unbleached enamel of the three and a significant response to a dual-cure activator; this is consistent both with the reduction observed here and with the low absolute values discussed above.

Saleem et al. [30] provide a particularly relevant recent human-enamel timing analogue, although their bonding substrate, restorative system, bleaching protocol, and mechanical test all differed from those used here: they bonded orthodontic brackets with a self-adhesive resin cement after 35% hydrogen peroxide bleaching and measured tensile bond strength, with SEM, EDX, and AFM characterization. They observed partial but incomplete recovery at 14 days, with bond strength remaining below the unbleached group. Agreement in direction across substantially different experimental configurations supports further investigation of persistent post-bleaching impairment, but does not establish that the same mechanism operates in both models.

The antioxidant literature addresses a different question: the meta-analysis of Rodríguez-Barragué et al. [33] found that natural antioxidants significantly improve immediate bond strength to bleached enamel, but the comparator in those studies is immediate bonding without pretreatment rather than a delayed-bonding protocol, so restoration of bond strength by antioxidants and a persistent deficit after waiting are not contradictory findings.

Several factors may account for the divergence from studies reporting a return to unbleached values within 1–2 weeks. Differences in peroxide concentration, bleaching protocol and cumulative exposure, adhesive generation and chemistry, application mode (etch-and-rinse versus self-etch), substrate preparation, storage medium, specimen geometry, the timing of testing relative to bonding, and the composite used may all contribute to the divergent findings among studies. The 40% formulation used here delivers a higher oxidative load than the at-home and low-concentration agents that dominate the pooled literature, so recommendations derived from lower-concentration regimens should not be extrapolated uncritically to high-concentration in-office bleaching. The present finding is therefore best regarded as a reason to re-examine, rather than to overturn, the two-week recommendation.

The numerically lowest mean SBS was recorded at 7 days rather than immediately after bleaching, which was unexpected because immediate bonding is commonly considered most susceptible to residual peroxide and oxygen. Because the three bleached intervals did not differ significantly from one another, this pattern should not be read as evidence of progressive post-bleaching deterioration; it may reflect changes during storage, random variation, or the absence of time-matched controls. Accordingly, reductions relative to the 14-day reference were observed at all three intervals, but the present study cannot establish their relative ordering or the trajectory of recovery over time.

The two composites were included principally as a robustness check, and the comparison between them is secondary to the main question. Because failure occurred predominantly at the adhesive–enamel interface, composite formulation would not be expected to exert a large influence on SBS, and no significant effect of material was detected despite the contrasting formulations tested. The condition effect was summarized across the two tested composites using estimated marginal means from the factorial model. No statistically significant material × condition interaction was detected; because sensitivity to small interaction effects was limited, however, material-specific heterogeneity cannot be excluded, and the absence of a detected interaction should not be interpreted as equivalence between the composites. The optical and polymerization characteristics of the single-shade material were not measured here.

Across all eight groups, the overall distribution of fracture modes was not statistically significant (*p* = 0.377), reflecting the small number of specimens per cell and the sparseness of the non-adhesive categories. Adhesive failure was nevertheless descriptively more frequent in the delayed bleached groups (38/40, 95%) than in the 14-day unbleached reference groups (13/20, 65%). Because this comparison was not prespecified and the study was not designed to investigate interfacial brittleness, the pattern should be regarded as hypothesis-generating. It could reflect impaired interfacial polymerization associated with residual oxidizing species, bleaching-related alteration of the enamel substrate, or both. No cohesive enamel failures were observed, which provides no evidence of gross enamel fracture under the tested conditions. Because interfacial degree of conversion and enamel mechanical properties were not measured, however, the present experiment cannot distinguish between adhesive-side and substrate-side mechanisms; the surface demineralization and increased porosity documented by EDX and AFM after bleaching [29,30] would be the relevant substrate-side change. Resolving these possibilities would require interfacial degree-of-conversion analysis together with mechanical characterization of the adjacent enamel.

Clinically, these data suggest that clinicians should be cautious about assuming that a conventional 1–2-week waiting period necessarily restores enamel bond performance to the level of unbleached enamel after high-concentration in-office bleaching. However, the finding requires confirmation in a specimen configuration yielding conventional absolute bond strengths before any revision of the recommended interval could be justified, and it should not be generalized to other bleaching formulations, adhesive systems, or application modes. Where restoration is required soon after bleaching, antioxidant pretreatment [34,35,36] or biomimetic remineralizing pretreatment [23], and removal of a thin superficial layer of bleached enamel [17], merit further investigation, although none was evaluated here.

The present study cannot define an optimal post-bleaching waiting interval. For elective adhesive treatment, the existing evidence supporting an approximately two-week delay remains the most defensible default [16]; the time-matched 14-day comparison reported here indicates, however, that restoration to the unbleached reference level should not be assumed for the specific bleaching and adhesive protocol tested. Evidence beyond 14 days remains insufficient to recommend a longer specific interval, and the present data cannot rank the immediate, 7-day, and 14-day conditions against one another. Where restorative treatment cannot be deferred, published in vitro evidence suggests that antioxidant pretreatment [33] and activator-assisted polymerization [26] warrant consideration and further clinical evaluation, but neither was tested here. These findings should accordingly not be used to prescribe a specific chairside protocol.

### 4.1. Waiting Versus Active Strategies for Restoring Bond Strength

Waiting is only one of four strategies proposed for restoring adhesion to bleached enamel, and the present data are best understood in relation to the others. Antioxidant pretreatment is the most extensively studied: sodium ascorbate, and more recently plant-derived agents such as grape-seed extract, green tea extract, bromelain and flavonoids, are applied to the bleached surface to consume residual oxidizing species before bonding [9,18,19,20,21,22,23,34,35,36]. The meta-analysis of Rodríguez-Barragué et al. [33] found a significant overall improvement in immediate bond strength with natural antioxidants, although the pooled estimate was heterogeneous and individual studies have reported failure to restore control-level values [35]. The advantage is that restoration can proceed in the same visit; the disadvantages are an added clinical step, application times commonly of 10 min or longer, and unresolved variation in agent, concentration, and vehicle.

Second, dual-cure and self-cure activators address the polymerization-inhibition mechanism directly rather than the substrate. Sivavong et al. [26] reported that a dual-cure activator significantly increased bond strength to bleached bovine enamel for two of three universal adhesives tested, including the adhesive used here. This is the alternative most directly relevant to the present protocol, since it adds an activator to an existing adhesive step rather than a separate clinical procedure, and it merits direct comparison against a waiting protocol within a single experiment. Third, mechanical removal of the outer 0.3–0.5 mm of bleached enamel restores bond strength by eliminating the affected substrate [17], at the cost of irreversible tissue removal that is difficult to justify on sound anterior enamel. Fourth, biomimetic remineralizing pretreatment has shown promise [23] but rests on a smaller evidence base.

None of these strategies was evaluated in the present study, and the comparison above is drawn from the literature rather than from the present data; that apparent SBS remained below the time-matched unbleached reference at 14 days does not establish that any alternative strategy would have produced reference-level bond strength. It does, however, indicate that the alternatives deserve to be tested against a waiting protocol, and not only against immediate bonding, which is the comparator used in most of the antioxidant literature.

### 4.2. Limitations

Experimental design. First and most importantly, time-matched unbleached controls were not included at the immediate and 7-day intervals, so comparisons of these two bleached conditions with the 14-day unbleached reference cannot separate an effect of bleaching from a possible effect of storage duration. The 14-day bleached and unbleached conditions were, in contrast, time-matched. The design therefore supports direct interpretation of the 14-day contrast but cannot establish the kinetics of recovery across the three post-bleaching intervals. Second, a single universal adhesive was evaluated in a single application mode, so the findings cannot be generalized to all universal adhesives or to their self-etch and selective-etch modes. Third, a single bleaching product and exposure protocol were used, so the conclusions are limited to 40% H_2_O_2_ under the tested protocol and cannot be generalized to all 35–40% peroxide formulations; the three-application exposure model also departed from the manufacturer’s stated application time.

Mechanical testing. The specimen configuration was non-conventional and yields absolute SBS values well below those obtained with standard geometries, so only relative comparisons are interpretable; the measurement is an apparent rather than a pure shear bond strength; and testing was performed immediately after mold removal, so neither the self-curing adhesive nor the composite had any period of post-cure maturation before loading. The degree of conversion at the interface was not measured. A micro-shear or micro-tensile configuration would have offered greater comparability with published absolute bond-strength values.

Biological and clinical model. Extracted teeth were used, and the bonding surfaces were ground with 600-grit paper before bleaching, so the substrate was prism-exposed rather than intact aprismatic enamel, whose oxidative response is not necessarily identical; residual enamel thickness after flattening was not measured instrumentally. Specimens were not thermocycled or otherwise aged, so the results reflect immediate bond strength rather than durability. Only premolars from donors aged 22–50 years were included, limiting generalization across tooth types and age groups. Finally, fracture-mode analysis used light stereomicroscopy rather than scanning electron microscopy, and classification was performed by a single examiner without a reliability assessment.

Statistical sensitivity. The design was powered a priori for the main effect of experimental condition. It was not designed for the material × condition interaction or for small differences among the post-bleaching intervals, and had approximately 80% power to detect a between-interval difference of 0.55 MPa, against observed differences of 0.14–0.37 MPa. The absence of significant differences among the three intervals is therefore inconclusive and must not be read as evidence that bond strength is stable between immediate bonding and 14 days; likewise, the non-significant interaction indicates an absence of detected interaction rather than equivalence between the two composites.

Future work should adopt a specimen geometry conforming to established protocols so that absolute values are interpretable, incorporate time-matched unbleached controls at every interval, extend the observation period beyond 14 days, include thermocycling, and evaluate the self-etch and selective-etch modes of universal adhesives, so that an evidence-based post-bleaching bonding interval can be defined across bleaching concentrations and adhesive systems. Such work should also pair mechanical testing with nanoindentation, degree-of-conversion measurement at the interface, and SEM or AFM imaging in the same specimen series, and should compare a waiting protocol directly against activator-assisted polymerization and antioxidant pretreatment, so that the choice between deferring and actively restoring adhesion can be made on comparative evidence.

## 5. Conclusions

In this in vitro model, apparent enamel SBS 14 days after 40% hydrogen peroxide bleaching remained significantly lower than that of the time-matched 14-day unbleached reference. Restoration to the unbleached reference level by 14 days should therefore not be assumed for the bleaching and adhesive protocol tested. Apparent SBS was lower in all three bleached conditions than in the 14-day unbleached reference condition, and H0_1_ was rejected.

Although the immediate and 7-day bleached conditions also showed lower SBS than the 14-day reference, the absence of corresponding time-matched unbleached controls prevents these comparisons from separating an effect of bleaching from an effect of storage duration, and from defining the kinetics of post-bleaching recovery. The study was, furthermore, not sufficiently powered to distinguish the relatively small differences observed among the three post-bleaching intervals.

No significant main effect of composite material and no significant material × experimental-condition interaction were detected, so there was insufficient evidence to reject H0_2_ or H0_3_; neither result should be interpreted as evidence of equivalence. Given the non-conventional specimen geometry and the low absolute SBS values, the results should be regarded as comparative in vitro evidence. Confirmation using time-matched controls at every interval, a conventional bond-test geometry, and combined mechanical and interfacial characterization is required before clinical post-bleaching bonding recommendations are modified.

## Figures and Tables

**Table 1:** Apparent shear bond strength (SBS) by composite material and experimental condition. Each specimen contributed one SBS value. SD, standard deviation; n, number of specimens.

Material	Experimental Condition	Group	Mean SBS (MPa)	SD (MPa)	n
Estelite Sigma Quick	Unbleached 14-day reference	G1	4.663	0.573	10
	Bleached, bonded immediately	G2	4.161	0.340	10
	Bleached, bonded at 7 days	G3	3.679	0.593	10
	Bleached, bonded at 14 days	G4	3.921	0.428	10
OMNICHROMA	Unbleached 14-day reference	B1	4.575	0.532	10
	Bleached, bonded immediately	B2	4.194	0.339	10
	Bleached, bonded at 7 days	B3	3.939	0.547	10
	Bleached, bonded at 14 days	B4	4.155	0.486	10

**Table 2:** Apparent shear bond strength (SBS) by experimental condition, pooled across both composite materials (n = 20 per condition), with Bonferroni-corrected comparisons against the unbleached 14-day reference condition. CI, confidence interval; SD, standard deviation.

Experimental Condition	Mean ± SD (MPa)	95% CI (MPa)	Reduction (%)	Mean Difference vs. Reference (95% CI), MPa	*p*
Unbleached 14-day reference	4.62 ± 0.54	4.40–4.84	NA	NA	NA
Bleached, bonded immediately	4.18 ± 0.33	3.96–4.40	9.6	0.44 (0.02 to 0.86)	0.034
Bleached, bonded at 7 days	3.81 ± 0.57	3.59–4.03	17.5	0.81 (0.39 to 1.23)	<0.001
Bleached, bonded at 14 days	4.04 ± 0.46	3.82–4.26	12.6	0.58 (0.16 to 1.00)	0.002

Reduction (%) is expressed relative to the unbleached 14-day reference condition. Confidence intervals for the condition means are unadjusted; confidence intervals for mean differences are Bonferroni-adjusted across the six pairwise comparisons. All were derived from the ANOVA error term (mean square error = 0.240, df = 72). Comparisons among the three post-bleaching bonding intervals were not significant: immediate versus 7 days, *p* = 0.120; immediate versus 14 days, *p* = 1.000; 7 days versus 14 days, *p* = 0.861.

**Table 3:** Two-way ANOVA of apparent shear bond strength by composite material and experimental condition.

Source	Type III SS	df	Mean Square	F	*p*	η^2^_p_
Corrected model	7.631	7	1.090	4.548	<0.001	0.307
Composite material	0.240	1	0.240	1.003	0.320	0.014
Experimental condition	6.978	3	2.326	9.703	<0.001	0.288
Material × condition	0.413	3	0.138	0.575	0.633	0.023
Error	17.259	72	0.240	NA	NA	NA
Corrected total	24.890	79	NA	NA	NA	NA

SS, sum of squares; df, degrees of freedom; η^2^_p_, partial eta-squared. The non-significant interaction indicates that no statistically significant difference in the pattern of the condition effect between the two composites was detected; the study was not designed or powered as an equivalence test, so this result does not establish that the two materials behave identically.

**Table 4:** Fracture-mode distribution by study group. Each group comprised 10 specimens, so percentages are based on a denominator of n = 10 throughout.

Group	Adhesive, n (%)	Cohesive in Enamel, n (%)	Cohesive in Resin, n (%)	Mixed, n (%)
G1	6 (60)	0 (0)	3 (30)	1 (10)
G2	8 (80)	0 (0)	2 (20)	0 (0)
G3	10 (100)	0 (0)	0 (0)	0 (0)
G4	10 (100)	0 (0)	0 (0)	0 (0)
B1	7 (70)	0 (0)	2 (20)	1 (10)
B2	6 (60)	0 (0)	3 (30)	1 (10)
B3	9 (90)	0 (0)	1 (10)	0 (0)
B4	9 (90)	0 (0)	0 (0)	1 (10)

Fisher–Freeman–Halton exact test across all eight groups: *p* = 0.377.

## Data Availability

The raw data supporting the findings of this study are available from the corresponding author upon reasonable request.

## Abbreviations

The following abbreviations are used in this manuscript:

ANOVA	Analysis of variance
CI	Confidence interval
H_2_O_2_	Hydrogen peroxide
ISO	International Organization for Standardization
SBS	Shear bond strength
SD	Standard deviation
SS	Sum of squares
